# Passively synchronized Q-switched and mode-locked dual-band Tm^3+^:ZBLAN fiber lasers using a common graphene saturable absorber

**DOI:** 10.1038/srep36071

**Published:** 2016-11-02

**Authors:** Chenglai Jia, Bhavin J. Shastri, Nurmemet Abdukerim, Martin Rochette, Paul R. Prucnal, Mohammed Saad, Lawrence R. Chen

**Affiliations:** 1 Department of Electrical and Computer Engineering, McGill University, Montréal, Québec H3A 0E9, Canada; 2 Department of Electrical Engineering, Princeton University, Princeton, NJ, 08544, USA; 3 Thorlabs, Inc., 56 Sparta Ave., Newton, NJ, 07860, USA

## Abstract

Dual-band fiber lasers are emerging as a promising technology to penetrate new industrial and medical applications from their dual-band properties, in addition to providing compactness and environmental robustness from the waveguide structure. Here, we demonstrate the use of a common graphene saturable absorber and a single gain medium (Tm^3+^:ZBLAN fiber) to implement (1) a dual-band fiber ring laser with synchronized Q-switched pulses at wavelengths of 1480 nm and 1840 nm, and (2) a dual-band fiber linear laser with synchronized mode-locked pulses at wavelengths of 1480 nm and 1845 nm. Q-switched operation at 1480 nm and 1840 nm is achieved with a synchronized repetition rate from 20 kHz to 40.5 kHz. For synchronous mode-locked operation, pulses with full-width at half maximum durations of 610 fs and 1.68 ps at wavelengths of 1480 nm and 1845 nm, respectively, are obtained at a repetition rate of 12.3 MHz. These dual-band pulsed sources with an ultra-broadband wavelength separation of ~360 nm will add new capabilities in applications including optical sensing, spectroscopy, and communications.

Known as the most stable heavy metal fluoride glass, ZrF_4_-BaF_2_-LaF_3_-AlF_3_-NaF (ZBLAN) is an excellent host for rare-earth ions. Rare-earth doped ZBLAN fiber supports lasing emission at a variety of wavelengths and has been successfully used to develop ultraviolet, visible, and infrared fiber lasers with continuous-wave (CW) and pulsed operation^1^,^2^. In particular, the ^3^H_4_→^3^F_4_ and ^3^F_4_→^3^H_6_ transitions in Thulium-doped (Tm^3+^) ZBLAN provide emission at ~1480 nm and ~1900 nm, respectively^1^. Situated in the S-band of the third communication window, fiber lasers operating at ~14xx nm are ideal pump sources for broadband Raman fiber amplifiers spanning the C-, L-, and U-bands^3^,^4^. Lying near one absorption peak of water, the 1480 nm wavelength also provides an effective means for water detection in various liquids. On the other hand, fiber lasers around the eye-safe 1900 nm region are useful for medical applications and chemical sensing. For example, by exploiting water absorption around 1900 nm, fiber lasers at this wavelength have been successfully applied for soft-tissue medicine and urinary stone ablation^2^,^5^. Moreover, lasers at 1900 nm are important for optical fiber communications which seek to exploit the transmission window from 1850 nm to 2100 nm^6^,^7^ and as pump sources to achieve emission further in the mid-infrared^8^.

A laser that operates simultaneously in two wavelength bands can provide enhanced functionality and new capabilities in sensing, instrumentation, and communications. Indeed, a number of dual-band sources with CW or pulse operation based on fiber lasers^9^, parametric oscillators^10^, and semiconductors (e.g., vertical cavity surface emitting lasers^11^) have been demonstrated. In particular, dual-band pulsed lasers are useful in multi-color pump-probe systems for time-resolved spectroscopy and multi-wavelength cavity ring-down spectroscopy^12^, or to develop transmitters operating simultaneously in two optical communication windows. Q-switching and mode-locking are the two main techniques for pulse generation in fiber lasers^13^. Q-switching is a modulation process of the quality factor Q of the laser cavity to produce high intensity pulses with typical durations ranging from μs to ns. On the contrary, mode-locking is a technique requiring balance of the intracavity dispersion and nonlinearity, and which induces a fixed-phase resulting in a single pulse with typical durations ranging from ps down to fs, and a repetition rate corresponding to the cavity round-trip time. Graphene has been rigorously employed in Q-switched and mode-locked fiber lasers to facilitate pulse generation^14^,^15^,^16^. Its unique electrical and optical properties enable it to be used as a saturable absorber (SA) with wavelength independent, broadband operation and fast response while its simplicity and flexibility of fabrication make it easy to be integrated in fiber lasers. Compared to other SAs such as doped bulk crystals, semiconductor SA mirrors, single wall carbon nanotubes, etc., graphene has distinctive advantages such as low saturation intensity, ultra-broadband operating wavelength range, high optical damage threshold, and ease of fabrication^15^.

In this work, we combine the use of a single gain medium—Tm^3+^:ZBLAN fiber—and a common graphene SA to support ultrabroad dual-band operation and demonstrate (1) a Tm^3+^:ZBLAN fiber ring laser with synchronized Q-switched pulses at wavelengths of 1480 nm and 1840 nm and a synchronized repetition rate from 20 kHz to 40.5 kHz and (2) a synchronous mode-locked Tm^3+^:ZBLAN fiber linear laser with full-width at half maximum (FWHM) pulse widths of 610 fs and 1.68 ps at wavelengths of 1480 nm and 1845 nm, respectively, and a repetition rate of 12.3 MHz. Previously, Wu *et al.*^17^ presented passive synchronization of Q-switched fiber lasers operating at 1060 nm with Yb-doped silica fiber and 1530 nm with Er:Yb-codoped silica fiber using a common graphene SA while Zhang *et al.*^18^ reported passive synchronization of two mode-locked fiber lasers at 1060 nm with Yb-doped silica fiber and 1540 nm with Er-doped silica fiber using a common single-wall carbon nanotube SA. Sotor *et al.*^19^,^20^ demonstrated a mode-locked fiber laser (without and with synchronization) operating simultaneously at 1565 nm with Er-doped silica fiber and 1944 nm with Tm-doped silica fiber using a common graphene SA. In all of these demonstrations, two separate gain media are employed, which often leads to increased complexity in implementation and laser design. Li *et al.*^8^ reported passively Q-switched pulses at ~3000 nm and gain-switched pulses at ~2000 nm pulses with Ho^3+^-doped fluoride fiber and a semiconductor SA mirror; however, the use of bulk optics eliminates the advantages of an all-fiber configuration. A graphene SA has also been applied to obtain Q-switched pulses at 1190 nm and 3000 nm with Ho^3+^-doped ZBLAN fiber^21^,^22^ while Q-switched pulses at 2780 nm and mode-locked pulses at 2800 nm were obtained with Er^3+^-doped ZBLAN fiber^23^,^24^. Here, we demonstrate for the first time the use of Tm^3+^:ZBLAN fiber to realize all-fiber dual-band pulsed laser sources. More importantly, we simplify the implementation of ultra-broadband dual-band sources using a single gain medium and one common graphene SA to achieve synchronized Q-switched and mode-locked operation. The pulsed sources are expected to provide enhanced functionality and capabilities in a number of applications from sensing to communications to instrumentation.

## Results

The dual-band passively synchronized Q-switched and mode-locked Tm^3+^:ZBLAN fiber lasers involve two separate cavities, one for each lasing wavelength, and a common path incorporating a graphene SA. The Q-switched laser is based on ring cavity configurations whereas the mode-locked laser is based on linear cavity configurations. Full details regarding the laser implementations are provided in the Methods section. In this section, we describe the performance and characteristics of the two lasers.

### Synchronous Q-switching

**Figure 1:**
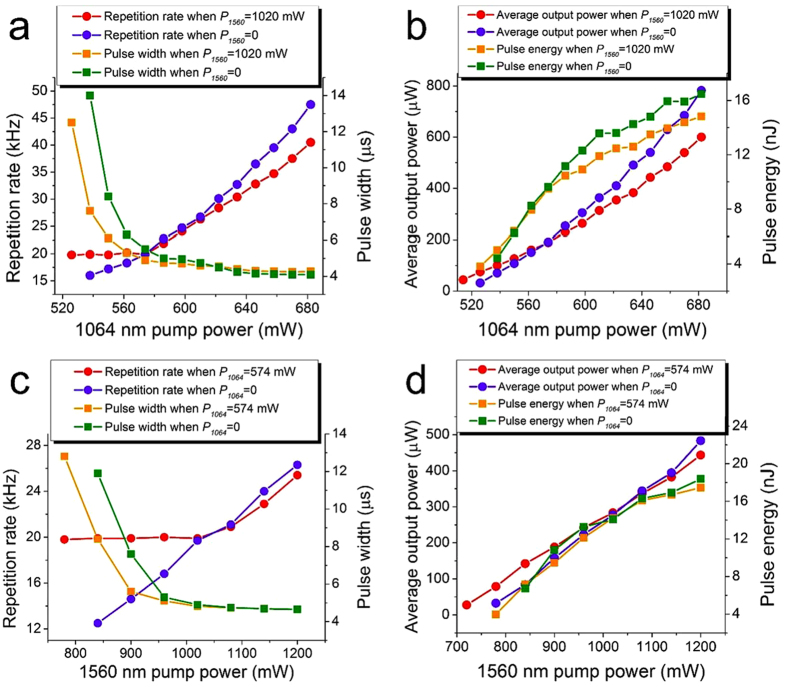
Characteristics of the dual-band synchronized Q-switched laser. (**a**) Repetition rate and pulse width and (**b**) average output power and pulse energy of the Q-switched pulses at 1480 nm when *P*_*1560*_ = 0 mW and 1020 mW; (**c**) repetition rate and pulse width and (**d**) average output power and pulse energy of the Q-switched pulses at 1840 nm when *P*_*1064*_ = 0 mW and 574 mW.

Figure 1 shows the measured repetition rate, pulse width, average output power, and calculated pulse energy of each ring cavity operating when the other cavity is either on or off. First, we turn off the 1840 nm cavity (the 1560 nm pump power is set to 0, i.e., *P_1560_* = 0 mW) and characterize only the 1480 nm cavity. CW lasing at 1480 nm is initiated when the 1064 nm pump (*P_1064_*) exceeds a threshold of ~526 mW. Stable 1480 nm Q-switched pulses are then established when *P_1064_* is increased to ~538 mW and is maintained up to ~682 mW with a repetition rate ranging from 16 kHz to 47.5 kHz at a rate of 219 Hz/mW. The corresponding pulse width first decreases exponentially from ~14 μs to ~4.2 μs, then keeps constant (at ~4.2 μs) due to the bleaching effect of graphene. The average output power increases linearly with a slope efficiency of 0.48% (such a low efficiency results from the relatively high cavity loss) while the pulse energy increases linearly and then saturates at a level of ~16 nJ, indicating that the graphene itself is almost saturated. When *P1064* = 682 mW, the average output power of the pulses at 47.5 kHz is 0.78 mW, while the corresponding pulse energy and peak power are 16.47 nJ and 3.77 mW, respectively. When *P_1064_* > 682 mW, no further Q-switching or stable pulsing operation is observed.

Pulse synchronization occurs when both cavities operate together and in particular, the cavity with a higher repetition rate dominates. For example, we set *P*_*1560*_ = 1020 mW power so that the cavity at 1840 nm produces Q-switching at 20 kHz, and then re-characterize the 1480 nm cavity. CW lasing at 1480 nm now starts when *P*_*1064*_ = 502 mW, which is lower than the threshold when the 1480 nm cavity operates alone, due to the change in the graphene transmission loss. As *P*_*1064*_ is varied from 526 mW to 574 mW, Q-switched pulses at 1480 nm appear now with a fixed repetition rate of 20 kHz (as opposed to increasing in repetition rate as is the case when *P_1560_* = 0 mW) and with pulse widths decreasing from 12.5 μs to 4.9 μs as shown by the red dots and orange squares in Fig. 1(a). Note that for this range of *P*_*1064,*_ both cavities produce Q-switched pulses at 20 kHz. As *P*_*1064*_ is increased further to 682 mW, the repetition rates of the Q-switched pulses at 1480 nm and 1840 nm both simultaneously increase from 20 kHz to 40.5 kHz, indicating that the repetition rate of the pulses from the cavity at 1840 nm follows that from the cavity at 1480 nm (as opposed to being fixed at 20 kHz in the absence of *P*_*1064*_).

We attribute synchronization to saturable absorption in the graphene alone. When pulses at the two wavelengths are sufficiently asynchronous (non-overlapping in time), both cavities operate independently and the individual repetition rates are controlled by the respective pump power (or equivalently, the corresponding pulse energy) as with typical Q-switching. This is due to the broadband and ultrafast response of graphene^14^,^15^. On the other hand, when the pulses at both wavelengths start to overlap temporally, the combined intensity on the common graphene saturable is higher (compared to when one wavelength operates alone) and this changes the operating dynamics/condition (balance between gain and loss) so that synchronization can be obtained.

Similar characteristics are observed in the 1840 nm cavity. In particular, we set *P*_*1064*_ = 0 mW and characterize the 1840 nm cavity. The threshold pump power for CW lasing at 1840 nm is *P*_*1560*_ ~720 mW. As *P*_*1560*_ is increased from 780 mW to 1200 mW, stable Q-switched pulses are observed with a repetition rate from 12.5 kHz to 26.3 kHz and a corresponding pulse energy and peak power up to 18.38 nJ and 3.71 mW, respectively. Next, we set *P*_*1064*_ = 574 mW to produce Q-switched pulses at 20 kHz at 1480 nm. As *P_1560_* is varied from 780 mW to 1020 mW, the Q-switched pulses at 1840 nm are now fixed at a repetition rate of 20 kHz. As *P*_*1560*_ is increased from 1020 mW to 1200 mW, the repetition rate of the Q-switched pulses at 1480 nm follows that of the pulses at 1840 nm.

**Figure 2:**
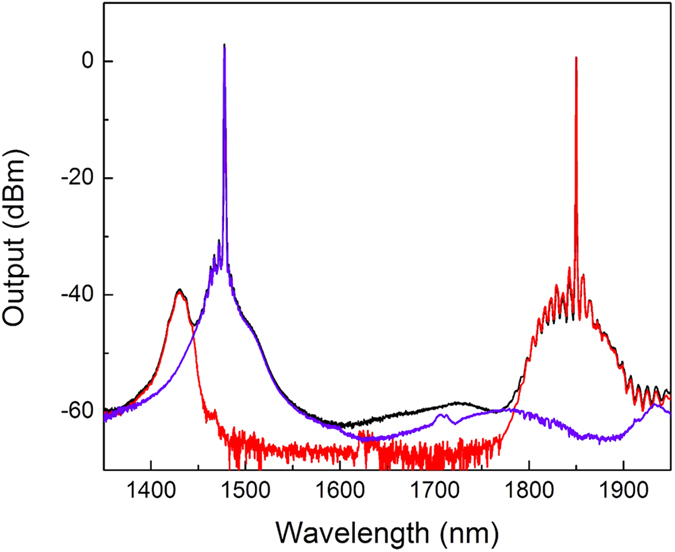
Output spectrum from the dual-band synchronized Q-switched laser. Simultaneous output wavelengths when *P*_*106*4_ = 574 mW and *P*_*1560*_ = 1020 mW (black), output at 1480 nm only when *P*_*1064*_ = 574 mW and *P*_*1560*_ = 0 mW (blue), and output at 1840 nm only when *P*_*1064*_ = 0 mW and *P*_*1560*_ = 1020 mW (red).

When the laser operates in synchronized Q-switched mode, tuning off one of the pump lasers will extinguish the corresponding output wavelength. Specifically, when we turn off *P*_*1560*_, there is no output at 1840 nm. Likewise, when *P*_*1064*_ is turned off, there is no output at 1480 nm. Figure 2 shows the output spectrum from the laser during synchronized Q-switched operation with *P*_*1064*_ = 574 mW and *P*_*1560*_ = 1020 mW and when only one cavity is active, highlighting that there is no residual output from the inactive cavity.

**Figure 3:**
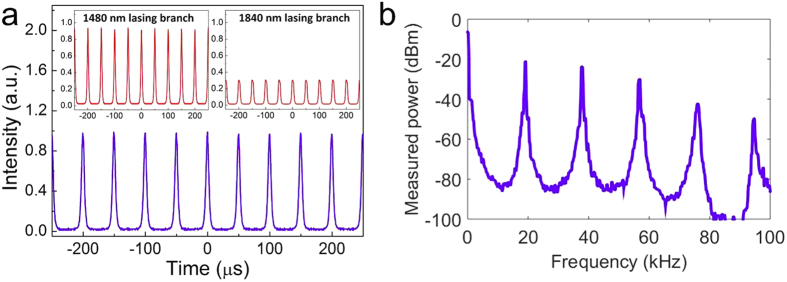
Time domain output and RF spectrum of the dual-band synchronized Q-switched laser. (**a**) Synchronized Q-switched pulses trains and (**b**) corresponding RF spectrum when *P*_*1064*_ = 574 mW and *P*_*1560*_ = 840 mW. The insets in (**a**) show the Q-switched pulses at the two wavelengths when the cavities operate alone.

Figure 3 shows the pulse trains in the time domain and the corresponding RF spectrum measured after the pulses at the two wavelengths are combined. As an example, when *P*_*1064*_ = 574 mW and *P*_*1560*_ = 840 mW, the combined and individual pulse trains coincide, demonstrating that they are completely synchronized. There is no amplification modulation in the pulses at either wavelength, indicating the absence of self-mode-locking effects on Q-switching. The RF spectrum shows a repetition rate of ~20 kHz with an RF signal-to-noise ratio (SNR) that exceeds 60 dB, further confirming the stable nature of the synchronized Q-switched pulses. Similar RF spectra are obtained when observing only the 1480 nm or 1840 nm output. Such ultra-broadband synchronized Q-switched pulses will be of great significance in the fields of nonlinear frequency conversion, chemical sensing, and spectroscopy^10^.

### Synchronous mode-locking

In order to improve the output efficiency and initiate dual-band mode-locking, two linear cavities with shorter cavity lengths and one common branch incorporating the graphene SA are utilized. Varying lengths of single mode fiber and an optical delay (ODL) are added so that the two cavity lengths can be tuned to obtain synchronous or asynchronous mode-locking. In both cases, we obtain stable fundamental mode-locked pulses at both 1480 nm and 1845 nm. In contrast to Q-switched operation, for mode-locking, we ascribe the synchronization to a combination of cross-phase modulation (XPM) as well as saturable absorption in the graphene.

**Figure 4:**
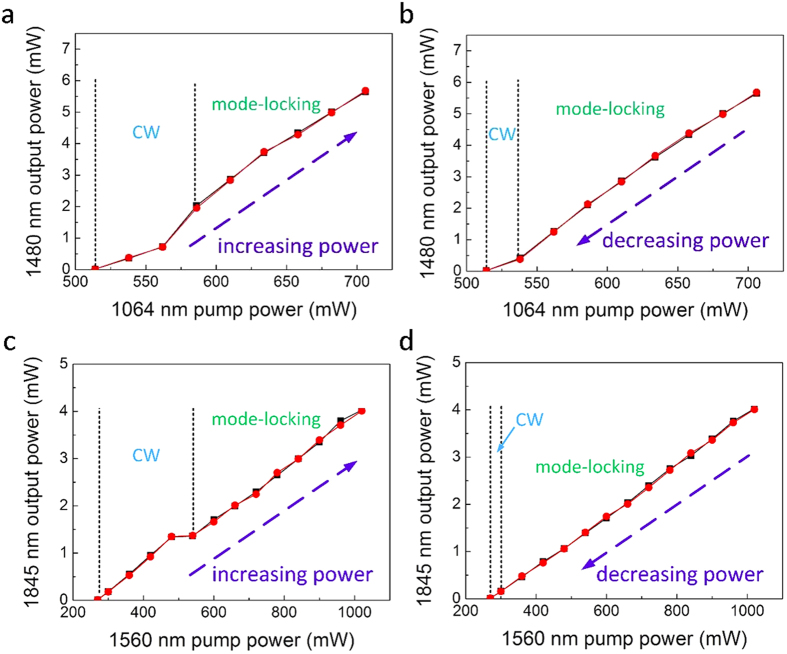
Characteristics of the dual-band laser when the cavity lengths are tuned for synchronized mode-locked operation. Measured output power at 1480 nm when the 1845 nm cavity is off (*P*_*1560*_ = 0 mW, red dots) and on (*P*_*1560*_ = 900 mW, black squares) for *P*_*1064*_ increasing **(a**) and decreasing (**b**). Measured output power at 1845 nm when the 1480 nm cavity is off (*P*_*1064*_ = 0 mW, red dots) and on (*P*_*1064*_ = 682 mW, black squares) for *P*_*1560*_ increasing (**c**) and decreasing (**d**).

Figure 4 shows the laser output power at 1480 nm and 1845 nm as a function of pump power [(a, c) show increasing pump power while (b, d) show decreasing pump power] when the ODL is tuned for synchronous operation. CW lasing at 1480 nm starts when *P*_*1064*_ = 514 mW. When *P*_*1064*_ is increased to 586 mW, the output power at 1480 nm increases significantly and its corresponding optical spectrum can be broadened with adjustment of the polarization controller (PC). As *P*_*1064*_ is increased further from 586 mW to 706 mW, stable fundamental mode-locking at 1480 nm is observed. To better protect the graphene SA, *P*_*1064*_ is controlled below 710 mW. When *P*_*1064*_ is decreased from 706 mW down to 538 mW, stable mode-locking is maintained. When *P*_*1064*_ is further reduced to 538 mW, CW lasing at 1480 nm reappears until the threshold of 514 mW. Similar to the 1480 nm loop, CW lasing at 1845 nm starts when *P*_*1560*_ = 270 mW; stable fundamental mode-locked operation at 1845 nm occurs from *P*_*1560*_ = 540 mW to 1020 mW. As *P*_*1560*_ is decreased from 1020 mW, mode-locking is continued until a power of 300 mW.

The two linear cavities operate independently. During simultaneous dual-band operation, adjustment of a PC in one cavity does not affect operation in the other. To verify further the independent operation of the two cavities, we re-measured the output powers at 1480 nm and 1845 nm when the other cavity is set to operate also in the mode-locking regime. The same evolution of output power vs. pump power (and hence laser operating regimes) are obtained whether or not the other cavity is active.

**Figure 5:**
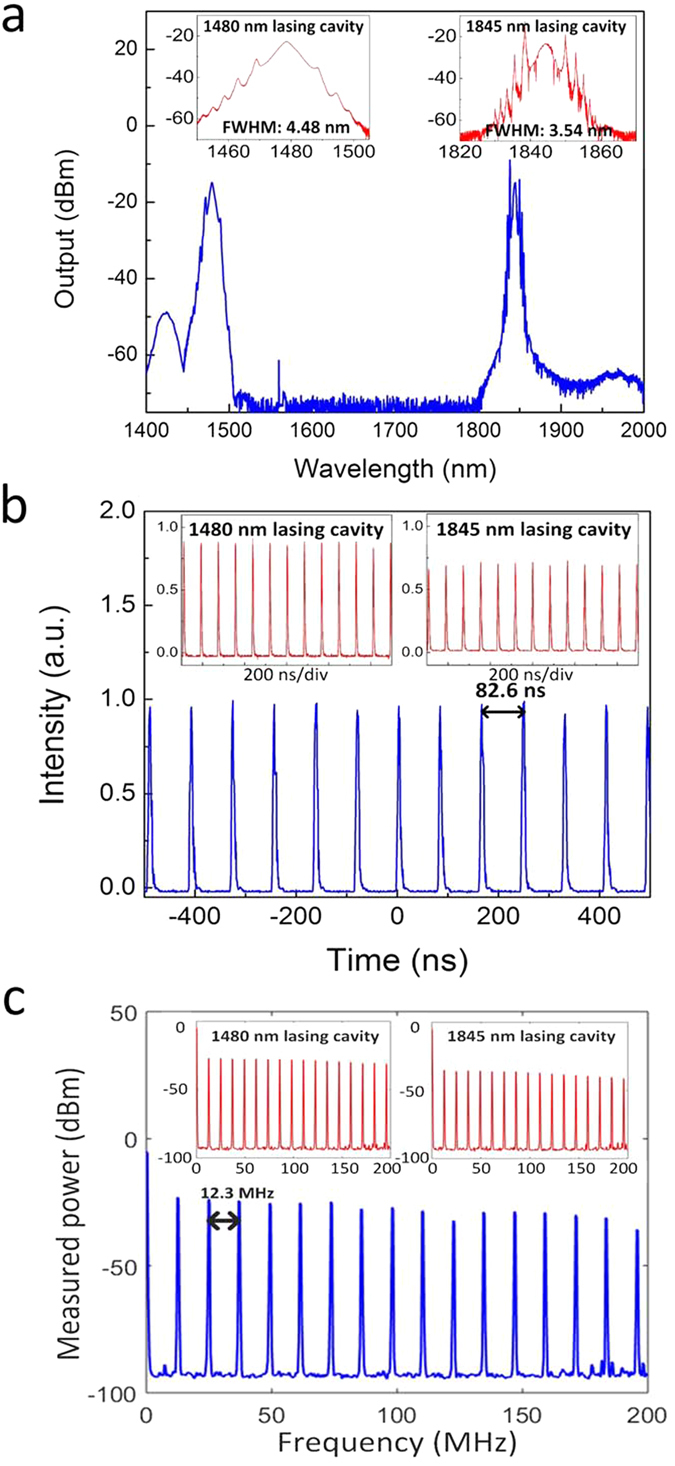
Output from, the dual-band laser when the cavity lengths are tuned for synchronized mode-locked operation with *P*_*1064*_ = 682 mW and *P*_*1560*_ = 900 mW. (**a**) Optical spectra, (**b**) combined temporal pulse train (the insets show the pulse trains at each wavelength operating independently), and (**c**) RF spectrum for the combined output pulses (the insets show the RF spectrum at each wavelength operating independently).

Figure 5(a) shows the typical optical spectrum at the output of the dual-band laser for synchronized mode-locked operation; the insets show the optical spectra in a 50 nm span centered at each wavelength with a resolution of 0.05 nm. The net dispersion of each cavity is anomalous so that the laser generates soliton-like pulses at both wavelengths; this is confirmed by the characteristic Kelly sidebands^17^. Note that the CW peak at 1560 nm originates from the unabsorbed pump power in 1845 nm cavity. The full-width half maximum (FWHM) bandwidths of the mode-locked pulses at 1480 nm and 1845 nm are 4.48 nm and 3.54 nm, respectively. The dips that appear near the pulses centered at 1845 nm are due to water absorption lines around 1900 nm.

Figure 5(b,c) show the mode-locked pulse trains in the time domain and the corresponding RF spectrum when *P*_*1064*_ = 682 mW and *P*_*1560*_ = 900 mW. From the individual pulse trains at 1480 nm and 1845 nm, or the combined pulse train, the pulses are separated by 82.6 ns, corresponding to a fundamental frequency of 12.3 MHz. The RF SNR exceeds 60 dB and indicates a high degree of synchronization between the mode-locked pulses at the two wavelengths.

**Figure 6:**
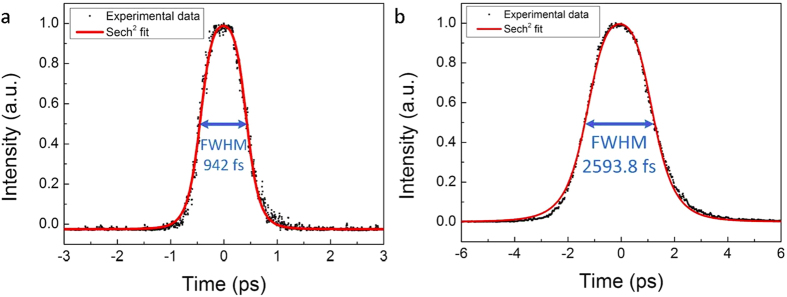
Autocorrelation traces of the output pulses from the dual-band laser when the cavity lengths are tuned for synchronized mode-locked operation. (**a**) 1480 nm (*P*_*1064*_ = 682 mW and *P*_*1560*_ = 0 mW) and (**b**) 1845 nm (*P*_*1064*_ = 0 mW and *P*_*1560*_ = 900 mW).

Figure 6 shows the measured autocorrelation of the mode-locked pulses at 1480 nm and 1845 nm along with sech^2^ fits. At 1480 nm, the pulse width after deconvolution is ~610.4 fs and considering the FWHM bandwidth of 4.48 nm, the corresponding time bandwidth product (TBWP) is 0.37. Due to the higher peak power required for the autocorrelator at 1845 nm, the pulses are first amplified using a Tm-doped fiber amplifier. At 1845 nm, the pulse width after deconvolution is ~1.68 ps and considering the measured FWHM bandwidth of 3.54 nm, the corresponding TBWP is 0.52 (note that pulse broadening occurs in the amplifier). Such dual-band mode-locked fiber lasers will be beneficial for developing optical transmitters in the S-band and around 2000 nm. Moreover, the lasers can be used as pump sources for supercontinuum generation at longer wavelengths^25^.

As shown in the Supplementary Note, similar results in terms of output power as a function of pump power and independent operation of the two cavities are obtained when the ODL and the cavity lengths are tuned for asynchronous operation (i.e., when the length of the 1480 nm cavity no longer matches that of the 1845 nm cavity). The main difference is the pulse width at 1845 nm, which is longer for asynchronous operation (the pulse widths at 1480 nm are comparable). We attribute this to the lack of XPM during asynchronized operation.

## Discussion

The dual-band Q-switched laser output is sensitive with the polarization state in each loop. For example, by adjusting the PCs in each laser cavity, the central wavelength can be tuned by ~5 nm and the average output power can be varied by ~20% for the same pump power; the corresponding pulse duration and repetition rate will also change by ~20%. For both cavities, the pulse durations and peak powers can be reduced by shortening the cavity length, using a higher dopant gain fiber, and optimizing the graphene sample by evanescent field interaction^26^.

For the dual-band simultaneous mode-locked Tm^3+^:ZBLAN fiber laser, no mode-locking output is observed if the graphene sample is placed between the Tm^3+^:ZBLAN fiber and the gold-tipped mirror to make light propagate through the SA twice in each oscillation. A linear cavity configuration is adopted instead of a ring cavity due to the relatively high cavity loss and limited pump powers. However, we believe that a ring cavity can support mode-locked operation if higher pump powers are used and the splicing loss between the Tm^3+^:ZBLAN fiber and SMF-28 is reduced. Finally, no wavelength selective elements are used in either laser; by incorporating tunable wavelength selective filters, wavelength tunable operation should be possible.

## Conclusion

We have demonstrated the use of a common graphene SA to implement (1) a passively synchronized Q-switched Tm^3+^:ZBLAN fiber laser at wavelengths of 1480 nm and 1840 nm with a synchronized repetition rate from 20 kHz to 40.5 kHz and (2) a synchronous mode-locked Tm^3+^:ZBLAN fiber laser operating simultaneously at wavelengths of 1480 nm and 1845 nm with pulse durations of 610 fs and 1.68 ps, respectively, and a repetition rate of 12.3 MHz. These two pulsed sources should offer enhanced capabilities in the various applications, including nonlinear frequency conversion, chemical sensing, spectroscopy, and communications.

## Methods

### Experimental setup

**Figure 7:**
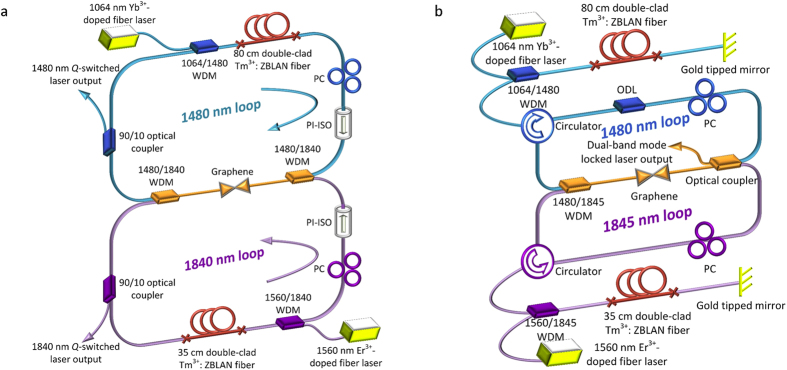
Experimental configurations of lasers. (**a**) Q-switched Tm^3+^:ZBLAN fiber ring laser and (**b**) mode-locked Tm^3+^:ZBLAN fiber linear laser.

Figure 7(a) shows the schematic of the passively synchronized Q-switched Tm^3+^:ZBLAN fiber laser. It comprises two ring cavities with one common branch incorporating the graphene SA. The gain fiber for lasing operation at 1480 nm (top loop) is an 80 cm length of Tm^3+^:ZBLAN fiber, whereas the gain fiber for lasing operation at 1840 nm (bottom loop) is a 35 cm length of the same fiber. The double-cladding Tm^3+^:ZBLAN fiber is doped with 8,000 ppm Tm^3+^, has an 8 μm core diameter, a 125 μm cladding diameter and is coated with 15 μm of mixed fluoroacrylate and acrylate. The Tm^3+^:ZBLAN fiber is coupled with SMF-28 fiber based devices through mechanical splices [represented by × in Fig. 7(a)], inducing ~2 dB loss per pair. The top ring cavity at 1480 nm is pumped by a 1064 nm Yb-doped fiber laser (*P*_*1064*_) via a 1064/1480 nm wavelength division multiplexer (WDM). A 1480 nm polarization independent isolator (PI-ISO) ensures the 1480 nm signal to propagate clockwise. The bottom ring cavity at 1840 nm is pumped at 1560 nm from an amplified laser (*P*_*1560*_) via a 1560/1840 nm WDM. A second PI-ISO ensures that the 1840 nm signal propagates counter-clockwise. Two optical couplers with a splitting ratio of 90/10 are used to extract the laser output from the two ring cavities (10% as the output ports). Two PCs are used to adjust intra-cavity polarization and to optimize the output in each cavity. Both signals are combined and separated via two 1480/1840 nm WDMs. The graphene film is deposited and sandwiched between two fiber connectors to form the SA^16^.

In order to improve the output efficiency and initiate dual-band mode-locking, two linear cavities with shorter cavity lengths and one common branch incorporating the graphene SA are used, as shown in Fig. 7(b). The same gain fiber and lengths of gain fiber used for the dual-band Q-switched laser are used again for the 1480 nm and 1845 nm cavities. Two gold-tipped mirrors serve as two reflectors to form one end of each cavity and two circulators designed for operation at 1480 nm or ~1845 nm are used to ensure unidirectional propagation and to allow for light oscillation in each linear cavity. Varying lengths of SMF-28 fiber and an ODL are added so that the two cavity lengths can be tuned to obtain synchronous (i.e., matched cavity lengths) or asynchronous mode-locking.

### Measurement methods

For dual-band Q-switched operation, the laser output is measured either (1) individually at the output from the 1480 nm or 1840 nm cavity directly or (2) after a 1480/1840 WDM coupler that combines the individual outputs from the two cavities. The first approach is used to characterize either cavity operating independently, i.e., in isolation of the other cavity, while the second allows observation of the dual-band output when both cavities are active. Note that the second approach also allows us to observe the output from one cavity when the other is turned off/inactive. For dual-band mode-locked operation, due to the configuration of the laser, the output is measured from the common branch of the output coupler only. Similar to the second measurement approach for Q-switched operation, we can observe the output from either cavity operating independently or with both cavities active.

For output power measurements, we use a power meter (Thorlabs, PM100D) along with a thermal power sensor (Thorlabs, S302C) with a wavelength range of 0.19–25 μm and a resolution of 1 μW. Note that all the pump power values shown in this paper are measured after WDM couplers.

The optical spectrum is measured by an optical spectrum analyzer (OSA, YOKOGAWA, AQ6375) with a scanning range from 1200 nm to 2400 nm and a resolution bandwidth of 0.05 nm. The temporal pulse trains are monitored by a combination of a 7 GHz photodetector (Newport, 818-BB-51F) and a 60 MHz oscilloscope (Agilent, 54621A). The RF spectrum is measured by an electrical spectrum analyzer (ESA, Anritsu, MS2668C) with a scanning range from 9 kHz to 40 GHz. The mode-locked pulses at 1480 nm and 1845 nm are characterized using different autocorrelators optimized for the two wavelengths (Femtochrome, FR-103XL for 1480 nm and Femtochrome, FR-103HS for 1845 nm).

## Additional Information

**How to cite this article**: Jia, C. *et al.* Passively synchronized Q-switched and mode-locked dual-band Tm^3+^:ZBLAN fiber lasers using a common graphene saturable absorber. *Sci. Rep.* **6**, 36071; doi: 10.1038/srep36071 (2016).

**Publisher’s note**: Springer Nature remains neutral with regard to jurisdictional claims in published maps and institutional affiliations.

## Supplementary Material

Supplementary Information
