# Supplementary material for: Passively synchronized Q-switched and mode-locked dual-band Tm3+:ZBLAN fiber lasers using a common graphene saturable absorber
Source: Sci Rep. 2016 Nov 2;6:36071. doi: 10.1038/srep36071 (PMC5090962; doi:10.1038/srep36071)
Supplement: Supplementary Information [file srep36071-s1.pdf]

Passively synchronized Q-switched and mode-locked dual-band Tm^{3+}:ZBLAN fiber lasers using a common graphene saturable absorber

Chenglai Jia,[1] Bhavin J. Shastri,[2] Nurmemet Abdukerim,[1] Martin Rochette,[1] Paul R. Prucnal,[2] Mohammed Saad[3], and Lawrence R. Chen[1*]

[1]Department of Electrical and Computer Engineering, McGill University, Montréal, Québec H3A 0E9, Canada.
[2]Department of Electrical Engineering, Princeton University, Princeton, NJ, 08544, USA.
[3]Thorlabs, Inc., 56 Sparta Ave., Newton, NJ, 07860, USA.

*Address correspondence to lawrence.chen@mcgill.ca.

Supplementary Note

When the cavity lengths are tuned for asynchronous operation (i.e., the length of the 1480 nm cavity is not matched to that of the 1845 nm cavity), the output characteristics from the laser are summarized below in Figs. 1 - 3.

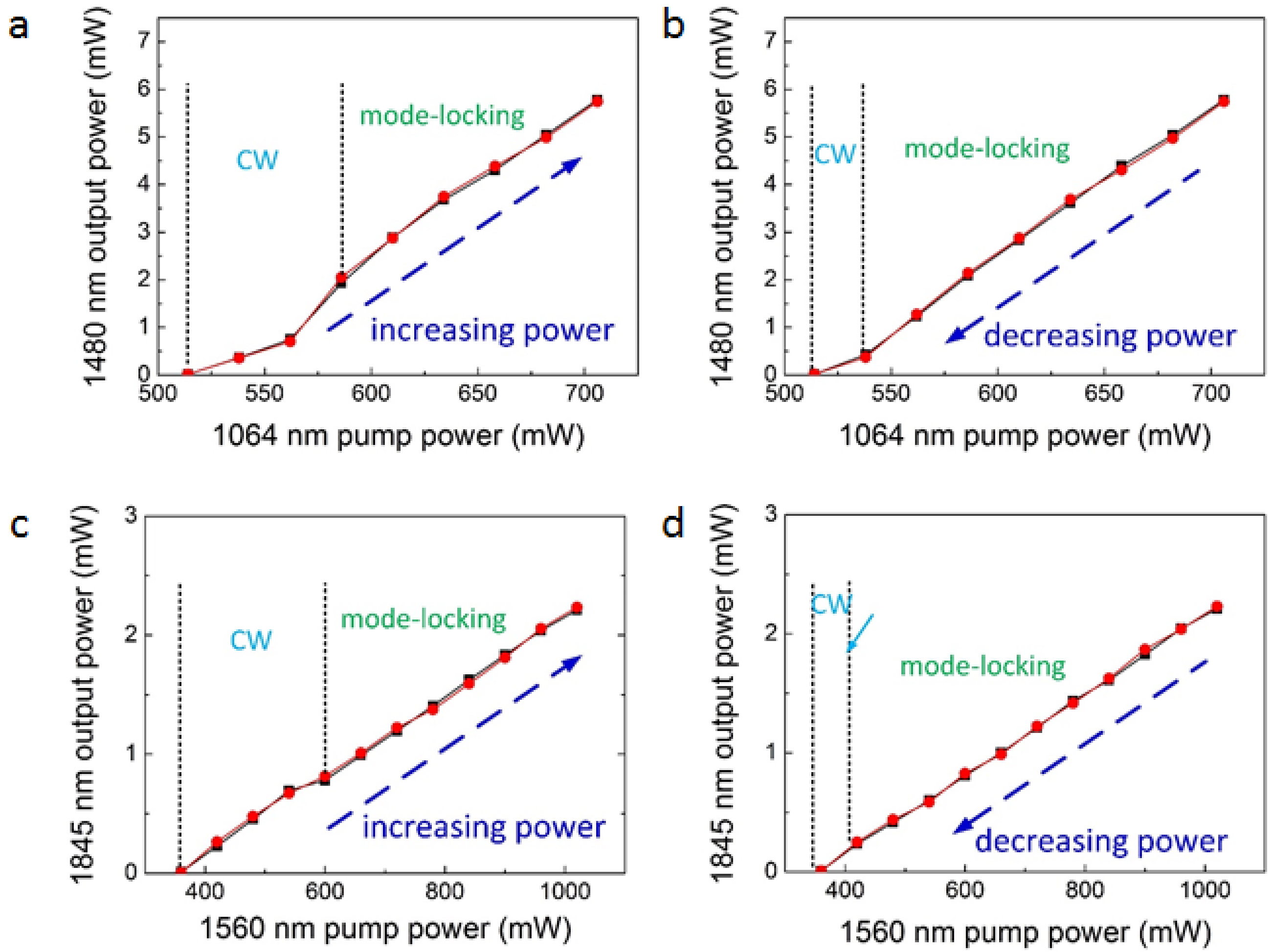

Fig. 1. Characteristics of the dual-band laser when the cavity lengths are tuned for asynchronous mode-locked operation. Measured output power at 1480 nm when the 1845 nm cavity is off (P_{1560} = 0 mW, red dots) and on (P_{1560} = 900 mW, black squares) for P_{1064} increasing (a) and decreasing (b). Measured output power at 1845 nm when the 1480 nm cavity is off (P_{1064} = 0 mW, red dots) and on (P_{1064} = 682 mW, black squares) for P_{1560} increasing (c) and decreasing (d).

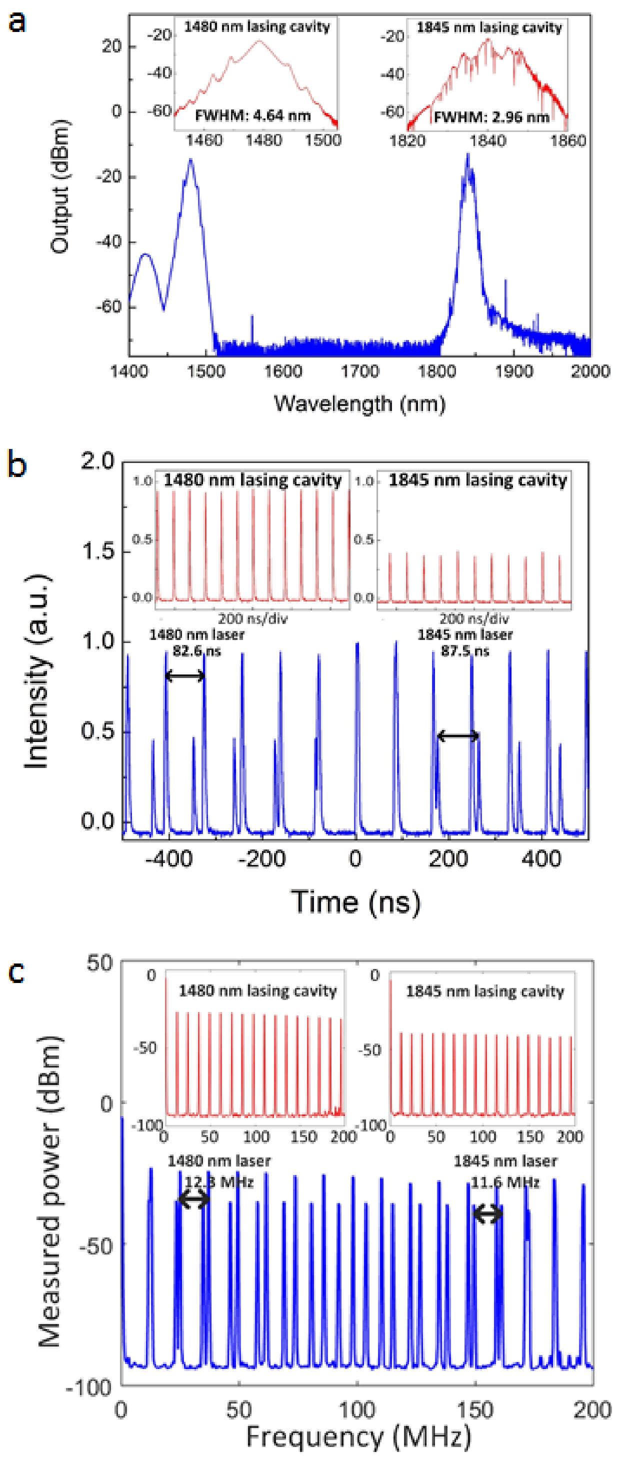

Fig. 2. Output from the dual-band laser when the cavity lengths are tuned for asynchronous mode-locked operation with P_{1064} = 682 mW and P_{1560} = 900 mW. (a) Optical spectra, (b) synchronized and combined temporal pulse train (the insets show the pulse trains at each wavelength operating independently), and (c) RF spectrum for the combined, synchronized output pulses (the insets show the RF spectrum at each wavelength operating independently).

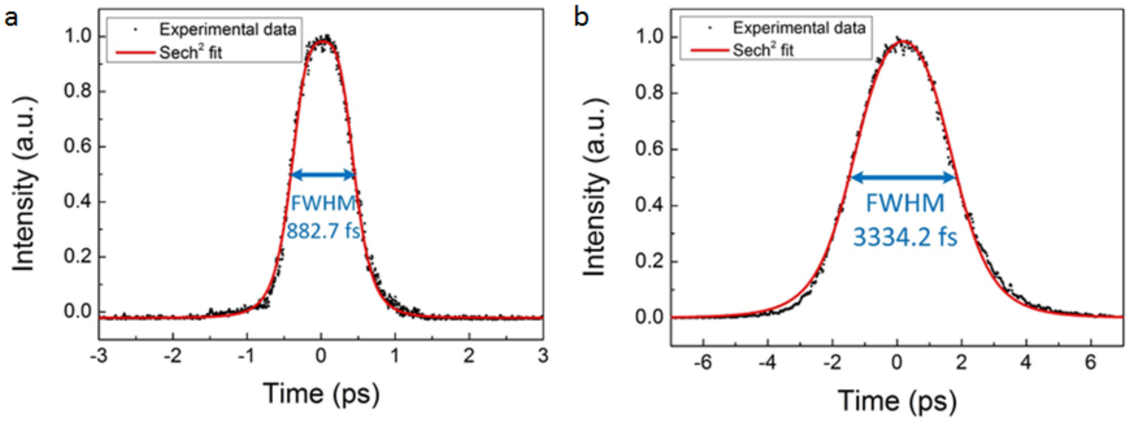

Fig. 3. Autocorrelation traces of the output pulses from the dual-band laser when the cavity lengths are tuned for asynchronous mode-locked operation. (a) 1480 nm (P_{1064} = 682 mW and P_{1560} = 0 mW) and (b) 1845 nm (P_{1064} = 0 mW and P_{1560} = 900 mW).
